# Phenolic contents-based assessment of therapeutic potential of *Syzygium cumini* leaves extract

**DOI:** 10.1371/journal.pone.0221318

**Published:** 2019-08-29

**Authors:** Rashid Ahmed, Muhammad Tariq, Maria Hussain, Anisa Andleeb, Muhammad Shareef Masoud, Imran Ali, Fatima Mraiche, Anwarul Hasan

**Affiliations:** 1 Department of Biotechnology, Mirpur University of Science and Technology, Mirpur, AJK, Pakistan; 2 Department of Mechanical and Industrial Engineering, College of Engineering, Qatar University, Doha, Qatar; 3 Biomedical Research Centre, Qatar University, Doha, Qatar; 4 Department of Bioinformatics and Biotechnology, Government College University, Faisalabad, Pakistan; 5 College of Pharmacy, Qatar University, Doha, Qatar; Nigde Omer Halisdemir University, TURKEY

## Abstract

*Syzygium cumini (S*. *cumini)* is an evergreen tropical plant that is well recognized for its therapeutic potential of common diseases. In this study, the therapeutic potential and biomedical application of *S*. *cumini* are assessed *in vitro* and *in vivo* to find its effectiveness for different complications. The methanolic crude extract of *S*. *cumini* leaves were screened for total phenolic and flavonoid content. *In vitro*, the DPPH scavenging assay, XTT assay, prothrombin and activated partial thromboplastin time were used to assess antioxidant, cytoprotective and thrombolytic activity of the *S*. *cumini* extract, respectively. The anti-inflammatory potential and the analgesic activity of the *S*. *cumini* extract were analyzed in rabbits by the Carrageenan induced paw edema method and the writhing method, respectively. Phytochemical analysis showed the presence of considerable amounts of total phenolic (369.75 ± 17.9 mg GAE/g) and flavonoid (75.8 ± 5.3 mgRE/g) content in the *S*. *cumini* extract. The DPPH assay demonstrated a higher antioxidant potential (IC-50 value of 133 μg/ml), which was comparable to the IC-50 of ascorbic acid (122.4 μg/ml). Moreover, the *S*. *cumini* extract showed a dose dependent cytoprotective effect against H_2_O_2_ treated bone marrow mesenchymal stem cells (BM-MSCs). *S*. *cumini* also possesses significant anticoagulant activity with a prothrombin time of 28.3 ± 1.8 seconds vs 15.8 ± 0.2 seconds of control, p<0.05. The leaf extract also demonstrated an analgesic effect in rabbits as indicated by the decrease in writhing (12.2 ± 1.7 control vs. 3.7 ± 0.6 treated) and anti-inflammatory activity in rabbits paw with a protection against inflammation of 64.1 ± 2.4%. Our findings suggest that the methanolic extract of *S*. *cumini* leaves has antioxidant, cytoprotective, anticoagulant, analgesic and anti-inflammatory properties, and therefore, can be applied for treating cardiovascular diseases and cancers.

## Introduction

Plants provide a large repertoire of phytochemicals such as polyphenols, flavonoids, carotenoids and vitamins, which are active ingredients of therapeutic drugs in modern age [1]. Natural plant components are associated with minimal side effects as compared to synthetic drugs and as such have gained much interest. More than 25000 phytochemicals have been identified to date [2] including more than 8000 different types of polyphenols for their therapeutic potential [3]. The therapeutic potential of plants are often attributed to their high phenolic content [4]. Recently, several studies have been carried out to ascertain the therapeutic potential of phytochemicals especially polyphenols and flavonoids against a number of ailments [5]. Specifically, the search for anti-inflammatory, antioxidant, anticancer and hypoglycemic agents from various origins including the fruits, spices, vegetables, medicinal herbs and teas has attracted remarkable attention [6, 7]. Thus, screening of traditional natural plant-based products is a logical approach in drug discovery.

Oxidative stress is one of main contributors of the pathophysiology of various pathological diseases [8]. Plants contain a diverse range of free radical scavenging molecules like terpenoids, phenolic compounds, vitamins and nitrogen compounds that have potent antioxidant activity [9]. Natural antioxidants have a variety of biochemical properties, including scavenging of free radicals, inhibition of reactive oxygen species (ROS) production and amendment of cellular redox potential. Inflammation stimulated in response to an infection has been associated with various diseases such as diabetes, cancer, atherosclerosis and hypertension [10, 11]. Non-steroidal anti-inflammatory drugs (NSAIDS) which are used for the treatment of inflammatory diseases are considered among the best-selling drugs worldwide despite the side effects associated with their use. Various plants with anti-inflammatory effects are used as alternative for the treatment of inflammation and thousands of reports exist on the anti-inflammatory properties of various plant tissue extracts [12]. Antidepressant drugs used as pain reliever in the cure of various chronic diseases have been traditionally obtained from plant based compounds [13]. There is a crucial need for active antithrombotic medicines to combat thrombotic diseases especially heart disease and cerebrovascular thrombosis [14]. Therefore, plant extracts can be used as alternative therapeutic agents for various medical issues due to the presence of diverse range of phytochemicals in them.

*Syzygium cumini (S*. *cumini)* commonly known as black plum, growing extensively in different agro-climatic countries possess several medicinal properties [6]. The extracts of various parts of *S*. *cumini* contains phytochemicals including tannins, anthocyanins, terpenes, flavanols and aliphatic-acids [15]. All parts of *S*. *cumini* are rich in polyphenols [16]. Both fruit and flowers of *S*. *cumini* are enriched in anthocyanins as cyanidin, delphinidin, peonidin, pelargonidin, petunidin, and malvidin [17]. Anthocyanins show anti-carcinogenic properties such as inhibition of tumor formation, induction of cell-cycle arrest and induction of apoptosis to cancer cells respectively [18]. The seeds of *S*. *cumini* contain rutin and quercetin while leaves have been reported to contain kaempferol, myricetin, quercetin and their glycosides. [19]. The plant has been reported to contain ellagic acid, triterpenoids, acetyl oleanolic acid, quercetin, isoquercitin, myricetin and kaempferol [20]. *S*. *cumini* possesses hypoglycemic, antimicrobial, hypolipidemic, anti-allergic, anti-inflammatory, cardio-protective, hepato-protective and antineoplastic properties [15, 16].

The antioxidant, analgesic, anti-inflammatory and other metabolic related features of *S*. *cumini* have extensively been reported of fruit and seed extracts, however, few studies exist on the applicability of *Syzygium cumini* leaves. In this study, we report the assessment of the antioxidant, anti-proliferative, anti-coagulant, anti-inflammatory and analgesic potential of *S*. *cumini* leaves extracts. The methanolic extract *Syzygium cumini* leaves was obtained and screened for total phenolic and flavonoid content. *In vitro* assessment of the phenolic extracts for antioxidant, anticoagulant and cytoprotective activities were also investigated. *In vivo* studies were conducted for anti-inflammatory and analgesic properties. Our findings demonstrate sufficient amount of phenolics and flavonoids in the leaf extracts of *S*. *cumini* and showed a remarkable level of antioxidant potential. The extracts of *S*. *cumini* demonstrated outstanding cytoprotective, anticoagulant activity and anti-inflammatory activities. Therefore, the present study suggests that *S*. *cumini* leaves extracts can be useful agents in therapeutic and biomedical applications, however, further studies will be needed to assess the molecular mechanism involved behind these activities on different kinds of cell lines and larger animals.

## Materials and methods

### Plant materials

Fresh leaves of *S*. *cumini* were collected from the local area of Mirpur, Azad Kashmir Pakistan at coordinates 34.22°N and 73.28°E, in spring season and were authenticated by an expert botanist in the Department of Biotechnology, Mirpur University of Science and Technology (MUST) AJ&K. The voucher specimen (MUST-728) was deposited in Department of Biotechnology. As this is not an endangered/protected species; there was no need to get approval for the use of leaves of *S*. *cumini* from the local environment protection department.

### Preparation of plant extract

The fresh leaves were shade-dried and cleansed properly to remove any extraneous material. The dried leaves were ground into coarse powder. The powdered plant material (300 g) was then refluxed with methanol (900 ml) and kept in the dark for 3 to 5 days with occasional stirring and shaking. The mixture was filtered using a cotton plug followed by filtration through a Whatman filter paper no. 1. The filtrate was dried at 50°C under reduced pressure, by rotary evaporator. The extract was preserved in an air-tight flask in a refrigerator as a crude extract.

### Determination of total phenolic and flavonoid content

The total phenolic content was determined using the Folin-Ciocalteu (FC) reagent method with slight modifications [21]. The concentration of polyphenols of the plant sample were determined using a calibration curve of standard reference gallic acid. Methanolic solutions (0.5 mL) of standard reference gallic acid at concentrations of 12.5, 25, 50, 100, 200 and 400μg/mL were added in 2.5 mL of 10% FC reagent and 2.5 mL of Na_2_CO_3_ solution (7.5%) and kept at room temperature for 1 h. The absorbance was measured at 765 nm by using UV-visible spectrophotometer. Samples were prepared in triplicates for each analysis and the mean values were taken. The same procedure was followed for single concentration of plant sample (200 μg/ml). The total phenolic content was determined as mg of gallic acid equivalent/g of dry tissue extract (mgGAE/g) by using formula: TPC = (X x V)/m; where TPC is total phenolic content, X is the gallic acid concentration in mg/ml; V is the extract volume used in ml and m is the weight of tissue extract in grams.

Total flavonoid content was assessed using spectrophotometric method with slight modifications [22]. Briefly, 1 ml of sample solution was added in 1 ml of ethanolic solution of AlCl_3_ and 1.5 ml of 5% sodium acetate solution followed by incubation at room temperature for 2.5 h. The absorbance was measured at 440 nm with UV-visible spectrophotometer. Rutin was taken as the standard while the total flavonoid content in extract was stated in terms of rutin equivalent (RE) mg/g of extract. The concentration of leaves extracts for this assay was 200 μg/ml. The experiment was carried out in triplicates.

### Antioxidant activity

The scavenging ability of the methanol extracts was assessed using the 2,2-diphenyl-1-picrylhydrazyl (DPPH) method adopted from a previous study [23]. 0.5 mM solution of DPPH (0.3 ml) was added to 0.5 ml of methanol extract at different concentrations (50–250 ug/ml) along with 3ml of absolute ethanol. The mixture was shaken strongly and placed in the dark for 60 min. The absorbance was measured against a blank at 517 nm. Ascorbic acid was used as a positive control. Three replicates were taken, and the scavenging ability was estimated by using following equation:

$$\%\;Inhibition=\frac{Absorbance\;of\;Control-Absorbance\;of\;Sample}{Absorbance\;of\;Control}\;X\;100$$

### Anticoagulant activity

The blood sampling was carried out from six healthy human donors voluntarily selected from Mirpur district of Azad Kashmir, Pakistan having no disease and taken no medicine for at least seven days prior to sampling. A written consent approved by the Research ethics committee (REC) of Mirpur University of Science and Technology was taken from each participant prior to blood sampling. The samples were taken in centrifuge tubes having 400 μL of 3.2% sodium citrate to avoid blood clotting followed by centrifugation @ 3000 rpm at 5°C for 15 min for plasma separation.

#### Prothrombin time (PT) test

The anticoagulant activity of plant tissue extract was assessed by the prothrombin time (PT) test described earlier by Mao et al., 2009 [24]. Briefly, the PT test was performed using a commercially available PT reagent kit (Singapore Biosciences, Singapore). Eighty microlitre of plasma was mixed with 20 μL of plant tissue extracts at the concentration of 1 μg/μL and then incubated at 37°C for 5 min followed by addition of 100 μL of pre-warmed thromboplastin reagent and the clotting time was recorded by a digital timer. Plasma without sample (with vehicle only) was used as control while Heparin was used as positive control.

#### Activated partial thromboplastin time (APTT) test

The activity of leaves tissue extract in the contact activation pathway and common coagulation pathway was measured by the APTT test based on the previously described method [24]. The APTT test is similar to the one described earlier and used to measure PT test. In the APTT test, 100 μl of the APTT assay reagent (Cephaloplastin) was used. The reaction was carried out at 37°C for 5 min. This was followed by incubation with 100 μl of 25 mM CaCl_2_ and the coagulation time was recorded.

### Animal studies

The cyto-protective activity of *Syzygium cumini* leaves extracts was performed on Bone marrow mesenchymal stem cells (BM-MSCs) of rats. Two young healthy inbred Sprague–Dawley (SD) rats were purchased from National Institute of Health (NIH), Islamabad, Pakistan. The rats were kept under controlled conditions at 22 ± 2°C and given standard diet.

Six to eight months old male rabbits weighing 1 to 1.5 kg were purchased from NIH, Islamabad, Pakistan. The rabbits were kept in separate cages at 22 ± 2°C and were provided with the balanced healthy diet and free mobility. All procedures on rats and rabbits were performed according to National Institute of Health, USA Publication No. 18–23, 1985 which is commonly used for the care and use of laboratory animals. Approval of all procedures and ethics for animal experimentation was sought from MUST Animal Care and Use Committee (MUST-ACUC) at Mirpur University of Science and Technology (MUST) through notification number MUST-2018-014.

#### Bone marrow mesenchymal stem cells (BM-MSCs) culturing

BM-MSCs were harvested from SD rats according to a previously reported protocol [25]. Briefly, the rats were sacrificed by cervical dislocation. The epiphyses were removed and whole bone marrow was flushed out from tibia and femur. The resulting suspension of bone marrow cells was centrifuged followed by re-suspension of cell pellet in Dulbecco’s modified Eagle’s medium (DMEM) enriched with 10% FBS and 1% antibiotic (100 μg/mL streptomycin and 100 U/mL penicillin). After harvesting from the bone marrow, the primary BM-MSCs were incubated with 5% humidified CO_2_ at 37°C. The cell culture media was changed on every 4^th^ day. When cells reached 80% confluence, they were sub-cultured and at passage 3 BM-MSCs were used in subsequent experiment.

#### XTT assay

The cytoprotective activity of *S*. *cumini* leaves extract was assessed by XTT assay [26]. BM-MSCs at passage 3 were trypsinized by using (1X Trypsin and EDTA). 1×10^4^ cells/well were plated in 96 well plates. The cells were placed in 5% CO_2_ incubator at 37°C by providing complete growth media containing 20% FBS (fetal bovine serum). At 80–90% confluency, the media was aspirated from the wells and BMSCs were washed twice with 1X PBS and treated with freshly prepared mixture of XTT. The plate was incubated in 5%CO_2_ incubator at 37°C for 24 h. The absorbance of the formazan product was analyzed by an ELISA plate reader at 450 nm and a reference wavelength of 630 nm to obtain sample signal (OD450-OD630). The percentage cell viability was calculated by the formula given below;

$$Cell\;Viability\;(\%)=\frac{Absorbance\;of\;Sample\;}{Absorbance\;of\;Control}X\;100$$

#### Anti-Inflammatory assay

The anti-inflammatory activity of *S*. *cumini* leaves extracts was evaluated using carrageenan-induced oedema method (acute *in vivo* model) in the hind paws of rabbits [12]. Rabbits were divided into five experimental groups, each group containing six rabbits (n = 6) and fasted for 18 h prior to the experiment. Three groups were administered the plant extract at three different concentrations of 25, 50 and 100 mg per kilogram of body weight as a suspension in 0.1% sodium CMC as vehicle. The rabbits of group three and four were given Ibuprofen (100 mg/kg) and Indomethacin (50 mg/kg), respectively as standard drugs. After 1 h of drug administration, 1% carrageenan (100 μL) in normal saline solution was injected in the sub-plantar area of the right hind paw of each experimental rat. After 1 h, 2 h and 3 h of the carrageenan injection, the reduction in paw volume was analyzed using plethysmometer. The percentage protection was calculated using the formula;

$$Protection\;(\%)=\frac{Reading\;of\;Control-Reading\;of\;Sample}{Reading\;of\;Control}X\;100$$

#### Analgesic activity

The analgesic activity was assessed by the acetic acid method reported previously by Ramírez et al. [12]. The animals were divided into 2 groups with 4 rabbits in each group. Control group rabbits were given saline solution while the treated group rabbits were given methanolic extract of *S*. *cumini* leaves at doses of 50 and 100 mg/kg dissolved in 5 ml saline solution and after 30 min 0.1% acetic acid was injected intra-peritoneal. The writhing (specific contractions of body) were observed randomly in rabbits and its frequency was counted for the next 20 min.

### Statistical analysis

All the data collected was analyzed by one-way analysis of variance, and differences among the means of groups by an unpaired, two-sided Student’s t-test. All the graphs were plotted in Microsoft Excel 2010 and OriginPro 2016. The values were calculated for their Mean and Standard Error of Mean (Mean ± SEM). The unpaired t-test was used to calculate p-value for each group to measure the degree of change occurring in parameters observed, for control group and test group as well. A p-value ≤ 0.05 was considered as significant value.

## Results

**Fig 1:**
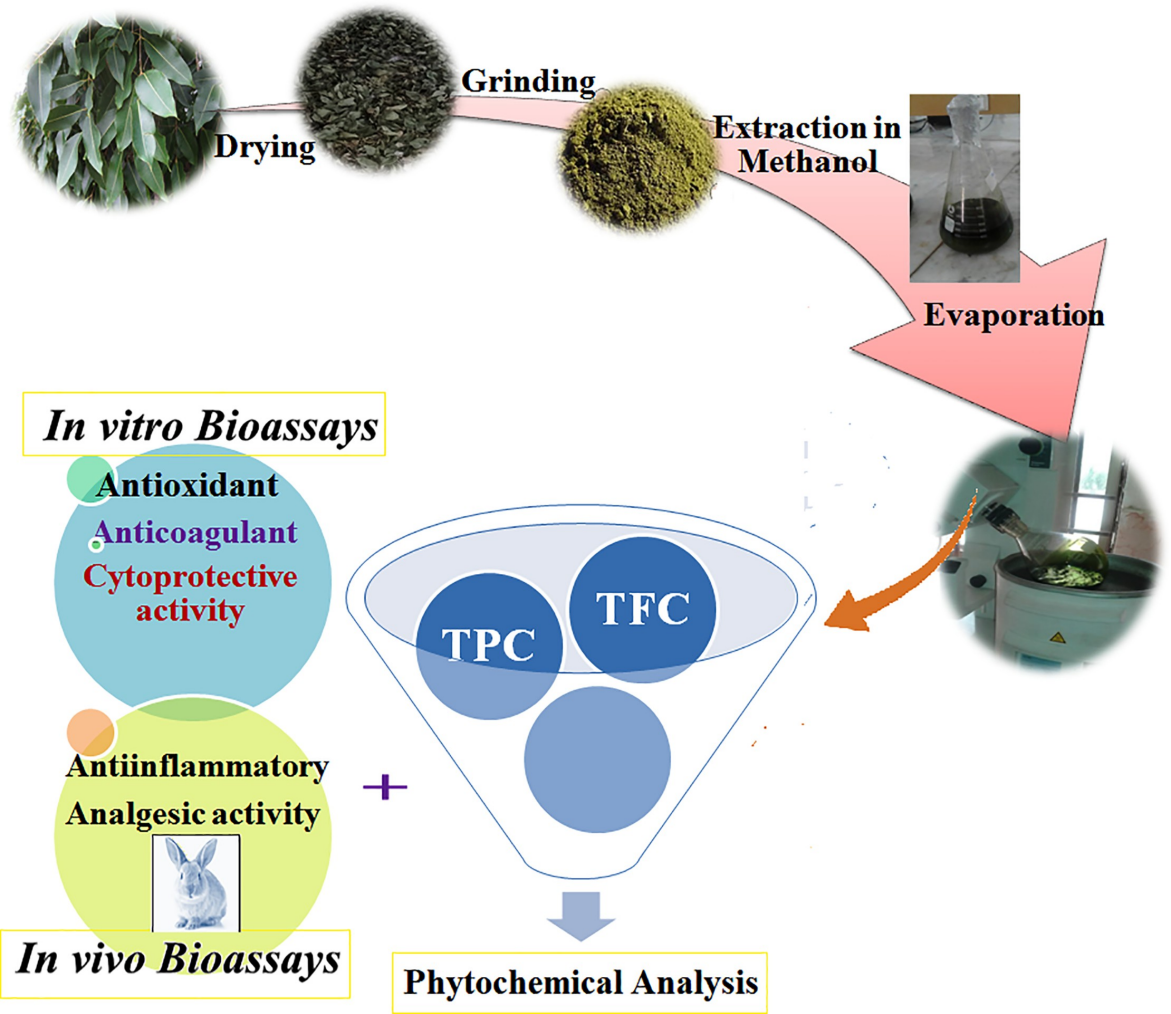
Graphical representation of experimental process for the whole study.

The therapeutic potential of *S*. *cumini* was evaluated by various assays for the determination of phenolic c and flavonoid contents. Further studies investigated the antioxidant, anti-coagulation, anti-inflammatory and analgesic efficacy as demonstrated in Fig 1. The results obtained from various assays both *in vitro* and *in vivo* showed a significant medicinal value of *S*. *cumini* methanolic leaf extracts.

### Total phenolic and flavonoid content

Total phenolic contents of *S*. *cumini* leaf extract as obtained by spectrophotometric method using Folin-Ciocalteu reagent are shown in Table 1. The linear equation obtained based on calibration curve of gallic acid was; Y = 0.004x + 0.1742 (R^2^ = 0.9931). The total phenolic contents measured for methanolic extract of leaves of *S*. *cumini* was 369.75 ± 17.9 mg GAE/g of plant extract.

**Table 1:** Total phenolic contents (TPC) and total flavonoid contents (TFC) of *Syzygium* leaves extract.

*Groups*	*Linear regression equation*	*Mean Absorbance of Plant Extract solution (Y)*	*Concentration of GAE in Plant sample (X)*	*Total contents calculated from equation = (X x V)/m*
Total Phenolic Content (TPC) mgGAE/g	Y = 0.004X + 0.1742	0.2482 ± 0.0036	18.5 ± 0.9	369.75 ± 17.9
Total Flavonoid Content (TFC) mgRE/g	Y = 0.0032X+ 0.0575	0.0696 ± 0.0009	3.79 ± 0.265	75.8 ± 5.3

Total flavonoid content in *S*. *cumini* leaf extract was determined by spectrophotometric method using Rutin as standard. The linear equation obtained from the calibration curve of rutin was; Y = 0.0032x + 0.0575 (R^2^ = 0.9851). The total flavonoid contents of *S*. *cumini* leaf methanolic extract calculated from the above equation was 75.8 ± 5.3 mgRE/g of plant extract. The presence of high content of phenolics and flavonoids in the leaves extract of *S*. *cumini* can be associated with the improved antioxidant, cytoprotective, anti-coagulant, anti-inflammatory and analgesic properties which are vital to reduce the risk from various diseases and offer multiple other health benefits due to their bioactivity and powerful antioxidant properties.

### Antioxidant activity

**Fig 2:**
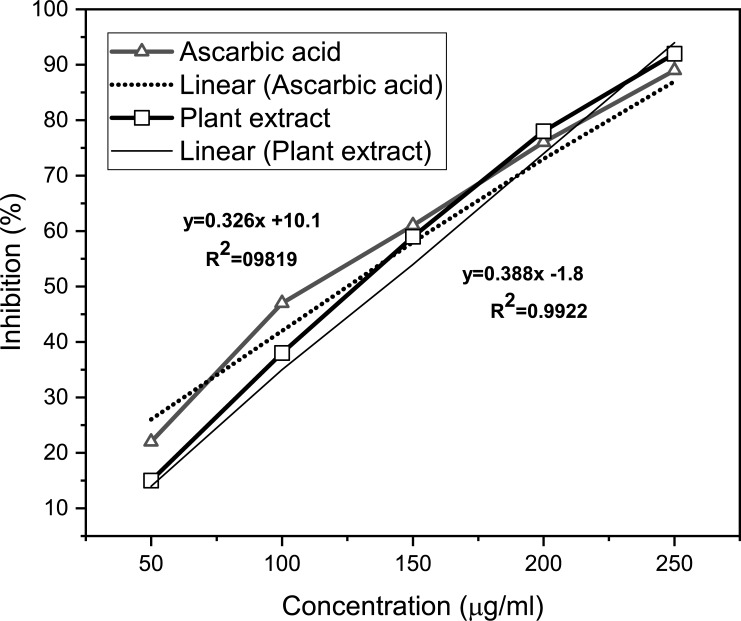
Antioxidant activity (DPPH radical scavenging activity) of *S*. *cumini* leaves extract and standard ascorbic acid. Each data point represents the mean of three experiments with standard error of the mean (Mean ± SEM, n  =  3).

The scavenging activity of leaves extracts and ascorbic acid was increased in a dose-dependent manner as shown in Fig 2. At concentrations of 50–250 μg/ml, the scavenging activities range of ascorbic acid and plant extract were 22 ± 2.3–89 ± 3.8% and 15 ± 4.5–92 ± 5.2% respectively. The IC-50 values were 122.4 μg/ml and 133 μg/ml for ascorbic acid and plant extract, respectively. Thus, the results indicated that the *S*. *cumini* leaves possess a high scavenging activity.

### Anticoagulant activity

**Fig 3:**
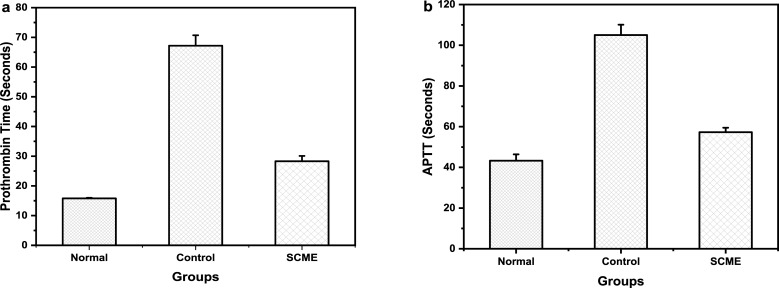
Anticoagulant activity. **(a)** Clot lysis by Prothrombin time (PT) assay *in vitro*, clotting time of *S*. *cumini* leaves extract was higher than normal but lower than control. **(b)** Activated partial thromboplastin time (APTT) assay *in vitro*. All readings are taken in triplicates and values are expressed as Mean ± SEM.

The S. *cumini* leaves extracts had significantly higher pro-thrombin time (28.3 ± 1.8 seconds) as compared to normal (15.8 ± 0.2 seconds) as shown in the Fig 3A (p-value < 0.05). The PT of control was 67.2 ± 3.5 seconds. Thus, the results indicated that *S*. *cumini* leaves extracts possess significant anti-coagulant activity.

The activated partial thromboplastin time for methanolic crude extract of *S*. *cumini* was assessed by using APTT assay reagent and. the results are shown in Fig 3B. The results depicted delayed APTT in plant sample (57.3 ± 2.2 seconds) as compared to normal (43.3 ± 3.1 seconds) while the APTT of control was 105 ± 5.1 seconds (p-value < 0.05).

### Cytoprotective activity

**Fig 4:**
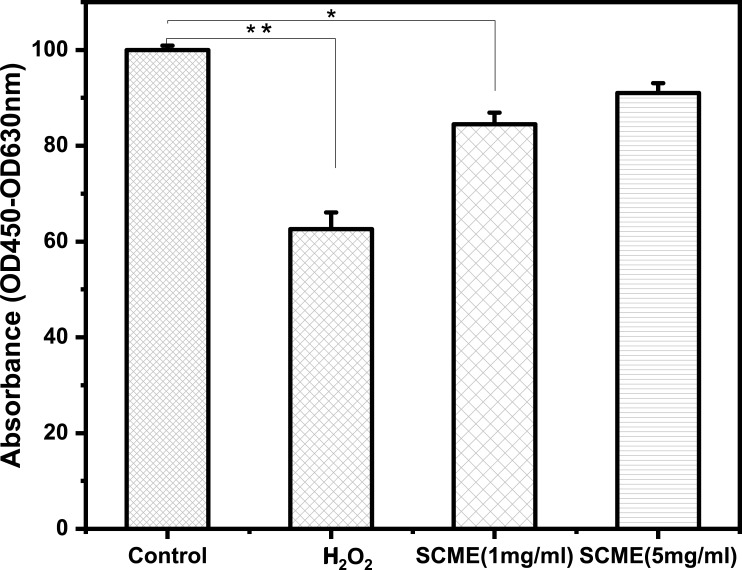
Cytotoxicity evaluation of *S*. *cumini* leaves extract by XTT assay on bone marrow derived mesenchymal stem cells (BM-MSCs). Cells were treated with plant extract at concentration of 1 mg/ml and 5 mg/ml for 24 h. *S*. *cumini* leaves extracts showed significant cytoprotective activity at increased dose of extract. Data is expressed as Mean ± SEM (n = 3).

The cytoprotective activity of leaves extracts of *S*. *cumini* on BM-MSCs obtained by the XTT assay are shown in Fig 4. BM-MSCs treated with hydrogen peroxide (H_2_O_2_) in the presence of leave extracts of *S*. *cumini* at concentration of 1 mg/ml and 5 mg/ml resulted in a concentration-dependent cytoprotective response (84.5 ± 2.4% at 1 mg/ml and 91 ± 2.1% at 5 mg/ml vs. 62.6 ± 3.5% H_2_O_2_ group) (Fig 4). Results indicated that with increasing concentration of plant samples, the cytoprotective activity was also increased.

### Anti-inflammatory assay

**Fig 5:**
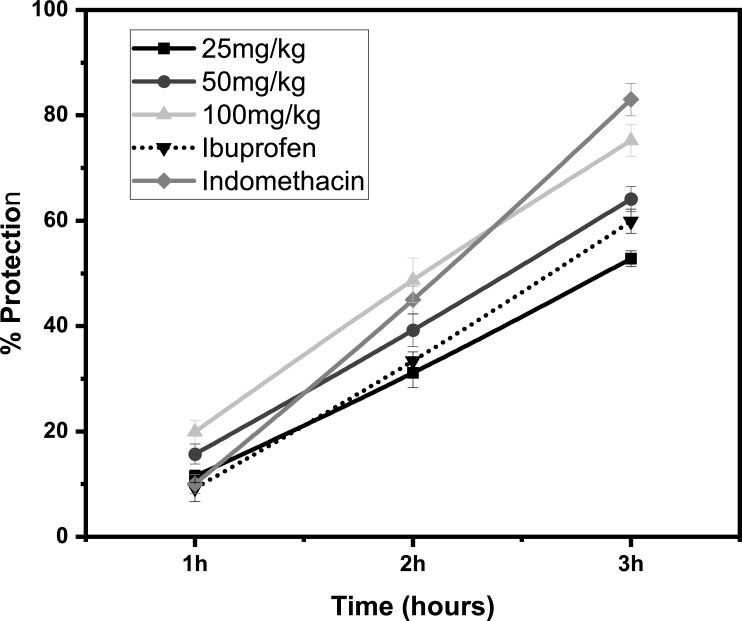
Anti-inflammatory activity of *S*. *cumini* leaves extract on carrageenan induced paw edema. Data is expressed as Mean ± SEM (n = 3).

The results of anti-inflammatory activity of methanolic crude extracts of *S*. *cumini* leaves determined by carrageenan-induced paw edema model of inflammation are shown in Fig 5. The results showed that the anti-inflammatory effect of the *S*. *cumini* was dose dependent as it increased from 52.8 ± 1.5% at 25 mg/kg to 64.1 ± 2.4 and 75.2 ± 1.9% for 25, 50 and 100 mg/kg respectively after 3 hr of carrageenan injection as shown in Fig 5. The results showed that leaves extract had higher anti-inflammatory potential (64.1 ± 2.4%) at 50 mg/kg as compared to standard COX-1 inhibitor Ibuprofen (59.9 ± 2.3%) and slightly lower compared to selective COX-1 and COX-2 inhibitor Indomethacin (83 ± 3.1%) after 3 h of induction of carrageenan. This considerable amount of anti-inflammatory activity as shown by leaves extract of *S*. *cumini* could play an important role in providing protection against various diseases that have high prevalence within populations such as rheumatoid arthritis, atherosclerosis and asthma.

### Analgesic activity

**Fig 6:**
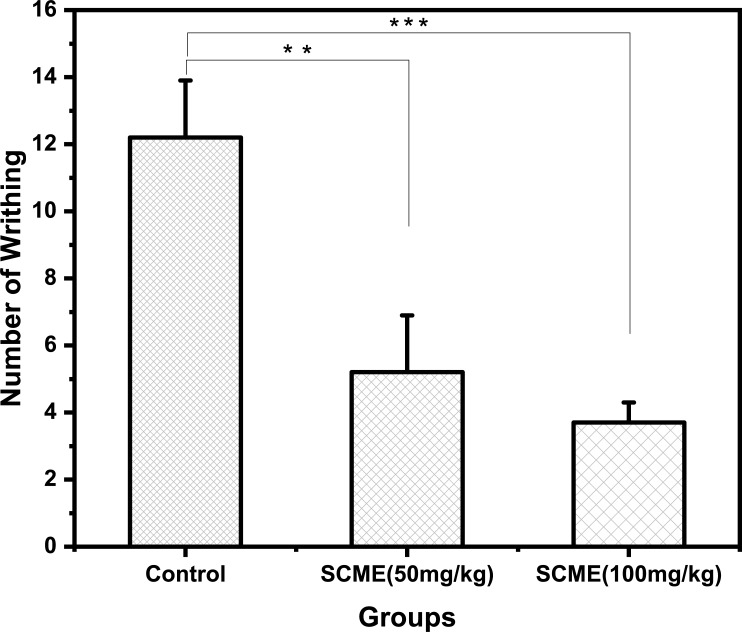
Analgesic effects of methanol extract of *S*. *cumini* leaves on acetic acid-induced writhing in rabbits. Values are taken in triplicates and expressed as Mean ± SEM (n = 3).

The analgesic activity of *S*. *cumini* leaves extract assessed by acetic acid induced writhing method is shown in Fig 6. The results show that the number of writhings resulted by the *S*. *cumini* leaves extracts treated group were 5.2 ± 0.9 and 3.7 ± 0.6 at the concentration of 50 and 100 mg/kg body weight, respectively. The writhing made by the control group was 12.2 ± 1.7 as shown in Fig 6. The administration of the methanolic leaves extract of *S*. *cumini* has significantly inhibited writhing response in animals in a dose dependent manner.

## Discussions

The phytochemical studies of *S*. *cumini* revealed the presence of saponins, carbohydrates, tannins, terpenes, sterols, volatile oil, resins, flavonoids, phlobatanins and balsam in the leaves and stem bark [27]. Phenols are important plant components due to their radical scavenging activity. The scavenging activity of phenols is attributed to their hydroxyl groups [28]. The phenolic content in the plants may directly contribute to the antioxidative activity. A previous study has suggested that polyphenolic compounds might have some inhibitory effects on carcinogenesis and mutagenesis in humans [29]. The leaves of *S*. *cumini* have been reported to have other bioactive compounds like gallic acid, citric acid, carotenoids, mallic acid, alkaloids and polyphenols, which are known to be effective for medicinal systems [3]. Methanolic extract of *S*. *cumini* leaves in the present study demonstrated good phenolic and flavonoid contents.

When plant components are proposed for use in clinical applications, taking their potential toxicity into consideration is also crucial [30]. The DPPH free radical is known as a stable free radical and considered as a widely used method for assessing free radical-scavenging potential of antioxidants. The powerful antioxidant property demonstrated by the tissue extracts of *S*. *cumini* was in fact due to high content of phenolics and flavonoids components [31]. Various pathologic situations generate free radicals including diabetes where inflammation is facilitated by neutrophils, macrophages and leukocytes through production of oxidative species [32]. The antioxidant results of the present study were supported by the previous work by Ruan et al. [6]. Therefore, the strong antioxidant and anti-inflammatory activities observed in this study might be due to the presence of high phenolic compounds in the extracts as displayed in Table 1.

To the best of our knowledge, no scientific study has so far reported the anticoagulant potential of methanolic extract of *S*. *cumini* leaves. This study reported is the first study to demonstrate the significant anticoagulant activity of methanolic extract of *S*. *cumini* leaves. Anticoagulation therapy rely on clot lysis for opening a blocked artery and to avoid permanent damage to the related tissues which is important for the treatment of clot-related diseases including thromboembolic strokes, pulmonary embolism, myocardial infarction and thrombosis [30]. In present study, the thrombolytic activity of *S*. *cumini* leaves extract was measured and compared with control. The experimental results showed significantly higher clot disruption activity by *S*. *cumini* leaves extract as compared to control. Anticoagulant effect of the methanolic extract is promising which suggests its potential use in thromboembolic disorders.

The cancer-protective effect of fruits and vegetables are related to their antioxidant activities which prevent DNA damage by scavenging free radicals. *S*. *cumini* reduced the tumor occurrence, tumor load and increasing level of gastric carcinomas [33]. It was reported that administration of the *S*. *cumini* extract (25 mg/kg body weight/day) inhibited benzo-a-pyrene- induced forestomach carcinogenesis [34]. Previously, the *S*. *cumini* seed extract has been reported to have anti-proliferative effects, which may be attributed to the high content of polyphenolics, flavonoids, quercetin, tannins and ellagitannins [33]. In the present study *S*. *cumini* leaves extract decreased the viability of BMSSCs *in vitro*. Significant cyto-protective activity of leaves extracts was observed at a concentration of 5 mg/ml as compared to those at lower concentration of 1 mg/ml. Thus, the earlier *in vivo* reports support our results.

The carrageenan induced model of inflammation has been widely used to evaluate anti-inflammatory potential of plant species in animal models. The earlier studies on ethanolic extracts [35] and methanolic extracts by [36] of the bark of *S*. *cumini* demonstrated that *S*. *cumini* extracts have strong anti-inflammatory properties. Another study evaluated the anti-inflammatory activity of methanol and ethyl acetate extracts of *S*. *cumini* seed in wistar rats using the carrageenan induced inflammation model, at the concentration of 200 and 400 mg/kg [37]. These studies suggested that both the bark and seed extracts of *S*. *cumini* have significant anti-inflammatory properties. These findings support our findings, which demonstrate the anti-inflammatory activity of the methanolic extract of *S*. *cumini* leaves using the carrageenan induced paw oedema model.

Acetic acid induced writhing response is an efficient method to assess peripherally acting analgesics [38]. Ayyanar and Subash-Babu reported that *S*. *cumini* leaves are rich in quercetin, myricetin, triterpenoids, acetylated flavonol glycosides, and tannin. These chemical components may be related to and act synergistically with the analgesic profile of *S*. *cumini* leaves extracts [39]. A recent study revealed that quercetin inhibited NO synthase in rat skeletal muscle [40]. The results of analgesic activity from the present study are in accordance with the previously reported work on *S*. *cumini* leaves antinociceptive activity on rodents [41]. Therefore, it is possible that the methanolic leaves extract of *S*. *cumini* may exert its peripheral analgesic action by interrupting the production, release or provoking the action of pain mediators and by inhibiting the local reaction caused by the pain at the target sites which result in significant analgesic effect. These bioactivities of *S*. *cumini* leaves extract can be linked to rich phenolic phytochemicals present in the extract.

## Conclusions

In the present work, the therapeutic potential of the leaves extracts of *S*. *cumini*, an evergreen tropical plant, is assessed through different bioassays in vitro and in vivo for its potential therapeutic and biomedical applications. The experimental work confirmed that a large amount of phenolics and flavonoids is embedded in the *S*. *cumini* tissues thus confirming the therapeutic usefulness of the parental plant. The manifestation of powerful antioxidant activity of *S*. *cumini* leaves extract can be related to the phenolic content present in the tissue extracts, which can provide protection from oxidative damages caused by free radicals and thus can prevent cancers. Similarly, the results obtained during analysis of anti-inflammatory activity of leaves extracts verified the beneficial effects by the traditional use of *S*. *cumini* plant extracts in the inflammation. Due to its significant anti-inflammatory potential, *S*. *cumini* leaves extracts have the ability to be used as therapeutic in various inflammatory conditions such as asthma, rheumatoid arthritis and atherosclerosis. Moreover, the *S*. *cumini* leaves extract showed significant analgesic activity, cytoprotective and anticoagulant activity which might be due to diverse range of bioactive polyphenols present in it.

The results of the study suggest that the methanolic extract of *S*. *cumini* leaves can be used as a supplementary or alternative herbal remedy for the treatment of inflammatory and blood disorders. The remarkable anticoagulant activity as observed during present work deserves further investigation involving constituents of the methanolic extract of *S*. *cumini* leaves that can be a good platform for the development of new class of anticoagulant drugs.
